# Supplementary material for: Exploring effects of response biases in affect induction procedures
Source: PLoS One. 2023 May 11;18(5):e0285706. doi: 10.1371/journal.pone.0285706 (PMC10174507; doi:10.1371/journal.pone.0285706)
Supplement: S1 Appendix — (DOCX) [file pone.0285706.s001.docx]

S1 Appendix

Modifications to the BMIS-VA during the experiment.

Several participants failed to recognise that the first line was an example. Many of them initially recorded their response to the first adjective on the example line, and recorded their responses to subsequent adjectives on the first side of the page on the line above the adjective rather than the one below, resulting in confusion when they reached the end of the first side and discovered an extra line. (Incorrectly filled out BMIS-VA questionnaires were accepted provided it was clear which responses were intended to relate to each adjective, and in unclear cases the participants were asked to clarify or provided with a fresh copy of the questionnaire.) The experimenter took to clearly and directly pointing out that the first line was an example scale when presenting the participants with the BMIS-VA, but this did not make any noticeable difference to the rate of mistakes. As a result, the scale was amended part way through the experiment to make the example line even more obvious, presenting the entire example scale within a box, and with a large “EXAMPLE” watermark printed across the middle of this box. This prevented further confusion among participants, and no alterations were made to the actual scale items. At the same time, one additional amendment was made to the questionnaire. To avoid requiring an additional short questionnaire, age and gender were now requested immediately above the BMIS-VA, on the same paper. This information was previously recorded on the consent forms, but these were also amended at the same time to ensure compliance with the EU General Data Protection Regulation (GDPR; 2018), which came into force during data collection.
