# Supplementary material for: Exploring effects of response biases in affect induction procedures
Source: PLoS One. 2023 May 11;18(5):e0285706. doi: 10.1371/journal.pone.0285706 (PMC10174507; doi:10.1371/journal.pone.0285706)
Supplement: S2 Appendix — (DOCX) [file pone.0285706.s002.docx]

S2 Appendix

Linear regression models for self-rated valence and arousal

**Table 1. Estimated coefficients, confidence intervals and associated p-values for linear model glm (Valence ~ Appraisal * Mood) using Neutral mood as reference category.**

| Valence Predictors | Estimates | 95% C.I. | p |
| --- | --- | --- | --- |
| (Intercept) | 19.62 | 5.38 – 33.86 | **.008** |
| Appraisal [Active] | -3.92 | -24.07 – 16.22 | .703 |
| Mood [Happy] | 31.38 | 11.24 – 51.53 | **.003** |
| Mood [Sad] | -47.48 | -67.63 – -27.34 | **<.001** |
| Appraisal [Active] * Mood [Happy] | -10.4 | -38.71 – 17.92 | .473 |
| Appraisal [Active] * Mood [Sad] | 25.15 | -3.34 – 53.63 | .086 |

**Table 2. Estimated coefficients, confidence intervals and associated p-values for linear model glm (Valence ~ Appraisal * Mood) using Happy mood as reference category.**

| Valence Predictors | Estimates | 95% C.I. | p |
| --- | --- | --- | --- |
| (Intercept) | 51 | 36.76 – 65.25 | **<.001** |
| Appraisal [Active] | -14.32 | -34.22 – 5.58 | .161 |
| Mood [Neutral] | -31.38 | -51.53 – -11.24 | **.003** |
| Mood [Sad] | -78.86 | -99.01 – -58.72 | **<.001** |
| Appraisal [Active] * Mood [Neutral] | 10.4 | -17.92 – 38.71 | .473 |
| Appraisal [Active] * Mood [Sad] | 35.54 | 7.22 – 63.86 | **.015** |

**Table 3. Estimated coefficients, confidence intervals and associated p-values for linear model glm (Arousal ~ Appraisal * Mood) using Neutral mood as reference category.**

| Arousal Predictors | Estimates | 95% C.I. | p |
| --- | --- | --- | --- |
| (Intercept) | -12.09 | -20.60 – -3.58 | **.006** |
| Appraisal [Active] | -5.03 | -17.06 – 7.00 | .414 |
| Mood [Happy] | -2.86 | -14.89 – 9.17 | .642 |
| Mood [Sad] | 3.13 | -8.91 – 15.16 | .611 |
| Appraisal [Active] * Mood [Happy] | 9.07 | -7.85 – 25.99 | .295 |
| Appraisal [Active] * Mood [Sad] | -4.05 | -21.06 – 12.97 | .642 |
