# Supplementary material for: Exploring effects of response biases in affect induction procedures
Source: PLoS One. 2023 May 11;18(5):e0285706. doi: 10.1371/journal.pone.0285706 (PMC10174507; doi:10.1371/journal.pone.0285706)
Supplement: S3 Appendix — (DOCX) [file pone.0285706.s003.docx]

S3 Appendix

Growth-curve analysis of FaceReader outputs

**Table 1. Estimated coefficients, confidence intervals and associated p-values for linear mixed-effect model (Valence ~ Appraisal * Mood * TimeStamp + TimeStamp | Participant) with Neutral mood as reference category.**

| Valence Predictors | Estimates | 95% C.I. | p |
| --- | --- | --- | --- |
| (Intercept) | 0.09 | -0.04 – 0.22 | .178 |
| Mood [Sad] | 0.02 | -0.16 – 0.21 | .803 |
| Mood [Happy] | 0.03 | -0.15 – 0.22 | .709 |
| Appraisal [Active] | -0.1 | -0.28 – 0.08 | .272 |
| TimeStamp | -0.03 | -0.08 – 0.02 | .204 |
| Mood [Sad] * Appraisal [Active] | 0.05 | -0.21 – 0.31 | .689 |
| Mood [Happy] * Appraisal [Active] | 0.07 | -0.18 – 0.33 | .565 |
| Mood [Sad] * TimeStamp | 0.03 | -0.05 – 0.10 | .461 |
| Mood [Happy] * TimeStamp | 0.06 | -0.01 – 0.13 | .091 |
| Appraisal [Active] * TimeStamp | 0.01 | -0.06 – 0.08 | .701 |
| (Mood [Sad] * Appraisal [Active]) * TimeStamp | 0.01 | -0.09 – 0.12 | .784 |
| (Mood [Happy] * Appraisal [Active]) * TimeStamp | -0.03 | -0.13 – 0.07 | .613 |

**Table 2. Estimated coefficients, confidence intervals and associated p-values for linear mixed-effect model (Arousal ~ Appraisal * Mood * TimeStamp + TimeStamp | Participant) with Neutral mood as reference category.**

| Arousal Predictors | Estimates | 95% C.I. | p |
| --- | --- | --- | --- |
| (Intercept) | 0.28 | 0.24 – 0.32 | **<.001** |
| Mood [Sad] | -0.02 | -0.07 – 0.03 | .471 |
| Mood [Happy] | -0.01 | -0.06 – 0.04 | .769 |
| Appraisal [Active] | 0.01 | -0.04 – 0.06 | .638 |
| TimeStamp | -0.02 | -0.05 – 0.01 | .122 |
| Mood [Sad] * Appraisal [Active] | -0.01 | -0.09 – 0.07 | .807 |
| Mood [Happy] * Appraisal [Active] | -0.05 | -0.12 – 0.02 | .178 |
| Mood [Sad] * TimeStamp | 0 | -0.04 – 0.04 | .849 |
| Mood [Happy] * TimeStamp | -0.04 | -0.08 – -0.00 | .048 |
| Appraisal [Active] * TimeStamp | -0.03 | -0.07 – 0.01 | .170 |
| (Mood [Sad] * Appraisal [Active]) * TimeStamp | 0 | -0.06 – 0.06 | .965 |
| (Mood [Happy] * Appraisal [Active]) * TimeStamp | 0.04 | -0.01 – 0.10 | .151 |
