# Supplementary material for: Exploring effects of response biases in affect induction procedures
Source: PLoS One. 2023 May 11;18(5):e0285706. doi: 10.1371/journal.pone.0285706 (PMC10174507; doi:10.1371/journal.pone.0285706)
Supplement: Attachment — Submitted filename: Response to Reviewers.docx [file pone.0285706.s004.docx]

Dear Editor,

Thank you for processing our submission and sending the helpful comments from the reviewers. Below we explain how we have addressed their concerns and suggestions. We also have amended the reference list as requested by the reviewers.

Sincerely,

Vera Kempe (for all authors)

Reviewer 1

In the introduction, when building the argument of self-reports against behavioural or physiological measures (line 72-74), should have more references to back up the argument (recent publications).

Response: We agree with Reviewer 1 that it would be important to provide more recent evidence. However, an extensive search confirmed our assertion, expressed in the draft in line 68, that this issue has faded from attention in more recent work. To support this, we have now included further references in lines 71 and 74 which have also been cited in a recent meta-analysis (Joseph et al, 2020) which are all of an earlier date.

When the authors state the need to investigate the biases in self-reports should have a paragraph explaining what has been done up to date in this area (line 74-77).

Response: Despite occasional concerns about biases in self-reports in the literature, our main point is that not much has been done in this area since the compelling meta-analysis by Westermann et al. from 1996. This is precisely what we are trying to remedy with our work. Our aim is clarified by the following sentence in line 68 ‘More recently, these discussions seem to have faded from attention even though the issues are far from settled, and most manipulation checks of AIPs still use self-reports.’ We hope the reviewer can agree with our concern that this issue needed to be taken up and scrutinised more thoroughly.

The same issue occurs in (line 84) when stating the need to use facial expressions. It should be an up-to-date paragraph stating the need to also include FACS in your investigation. Even though after proposing using FACS, you show examples, the build-up to find the need or gap should be further explained and explicit, especially if you then expose the positives and several negative reasons for using physiological indicators for this kind of research which is latter confirmed in the results.

Response: We thank the reviewer for pointing out the lack of clarity on our motivation for focussing on FaceReader. We have now added a sentence in line 89 to this effect, which reads ‘However, human coding of facial expressions using FACS is costly as it requires extensive certified training of the coders (12,13).‘ We feel that adding another paragraph discussing FACS would unduly lengthen the paper and divert focus but we are happy to take editorial guidance on this.

The study is well presented, with relevant references that support the study design. There is a “NOTE” in line 195- 220 that explain different outcomes of possible results that are redundant and without references to back up the claims; maybe this should go in the discussion?

Response: We thought it is important to justify in the Introduction why we are employing a two-tailed hypothesis; we fear it would create confusion for the reader to leave this important consideration for the Discussion. We agree that justification for the hypothesised outcomes would gain in persuasiveness if motivated by previous work so we included references to the literature on the link between emotions and cognitive load and to the measurement effects in the field of emotion research.

METHOD:

Four years to collect the data? please explain

Response: We understand the reviewer’s concern about our data collection procedure but data collection had to be interrupted due to personal circumstances of one of the authors.

Reviewer 2

The manuscript is clearly written and well-motivated. I think this study is a useful incremental contribution to the literature and ongoing discussion of the validity of AIPs. I would encourage the authors to consider discussing how demand effects may be moderated by experiment presentation (in other words, is it possible that active appraisal changes how participants view the goals or demands of the experiment?). However, I do not believe this request is sufficient to waylay publication.

Response: Wе thank Reviewer 2 for the positive evaluation of this work. We agree that it is possible that participants in the Passive Exposure condition are subject to demand characteristics in the sense that their mood reporting is affected by an appraisal of the images in addition to a reporting of their affective state. However, this would mean that in the Active Appraisal condition participants have already completed their appraisal when asked to report on their mood indicating the genuine effect of the mood induction above and beyond any appraisal-related demand characteristics. We have now clarified this in the Introduction in line 203-204.
